# Nutritional and biochemical profile and bioactive properties of four Greek local landraces of onion

**DOI:** 10.3389/fpls.2026.1743587

**Published:** 2026-02-05

**Authors:** Nikolaos Polyzos, Daiana Almeida, Ângela Fernandes, Tânia S. P. Pires, Tayse F. F da Silveira, Lillian Barros, Spyridon A. Petropoulos

**Affiliations:** 1 Department of Agriculture, Crop Production and Rural Environment, University of Thessaly, Volos, Greece; 2 CIMO, LA SusTEC, Instituto Politécnico de Bragança, Bragança, Portugal

**Keywords:** Alliaceae, *Allium cepa* L., antimicrobial properties, antioxidant activity, fatty acids, macronutrients, nutritional value, phenolic compounds

## Abstract

**Introduction:**

The preservation of genetic diversity of horticultural crops is essential for modern agroecosystems that face pressure from climate change and the intensification of crop production.

**Methodology:**

In the present work, the nutritive value, chemical profile and bioactive properties of four Greek onion local landraces were investigated for the first time. The tested genotypes included three local landraces originated from the region of Thiva, one landrace collected from Evoia, as well as one commercial cultivar which was used as the control treatment. All the genotypes were assessed in terms of nutritional value, chemical composition and bioactivities.

**Results and discussion:**

Our results indicate a high variability in the nutritive value and the chemical profile of the studied genotypes, while varied bioactive properties were also recorded. The bulbs of the local landrace ON5 were the richest in polyphenols and quercetin in particular, a finding which was also accompanied by the highest antioxidant activity. On the other hand, the commercial cultivar was the richest in total and individual free sugars, as well as in total dietary fibers and total organic acids, results which indicate a sweeter taste and higher pungency. In conclusion, the studied landraces showed promising properties and they could be further valorized through the implementation of breeding programs aiming to produce new elite onion genotypes with high nutritional and bioactive potential.

## 1 Introduction

Onion (*Allium cepa* L.) is a bulbous plant of the Liliaceae family, which includes more than 3700 species (Sansan et al., 2024). According to literature reports, onion has been acknowledged as one of the oldest cultivated plants, whereas Central Asia and more precisely certain areas of Iran and Pakistan are considered the main centers of origin of the species (Amare, 2020; Arena et al., 2024). Based on FAOSTAT data, the annual total production in Europe for the 2023 calendar year was 9.877.287,08 tones, harvested from around 319.198 ha with average yield of 30.944,1 kg/ha (FAOSTAT, 2024). In Greece, onion cultivation is very important and covers an area of 3.130 ha with an average yield of 34.840,3 kg/ha and a total production of 109.050 tones (FAOSTAT, 2024). Since ancient times, the onion has been utilized for culinary and medicinal properties throughout the centuries and nowadays both the fresh leaves and bulbs are the main ingredients used in many recipes providing a district flavor and aroma in various dishes (Sagar et al., 2022; Kant Upadhyay, 2023).

The fresh leaves and dry bulbs can be used in cooked dishes and salads, as condiments or spices, in raw salads, while they can be used after processing as pickles, powders, pastes and many more (Chakraborty et al., 2022). The skin of bulbs which is usually discarded in domestic and industrial processing is also a valuable by-product that can find food and pharmaceuticals applications through the extraction of bioactive compounds or the incorporation in functional bakery products (Piechowiak et al., 2020). In addition, the medicinal properties of onion have been widely acknowledged, since the species has been recognized in several ethnopharmacological studies as an effective remedy against many diseases namely gastrointestinal disorders, throat infection and hepatitis, whereas tea of onion presented severe properties against arthritis, cholera, common cold, fever and headache (Corzo-martı et al., 2007; Prima et al., 2023; Elattar et al., 2024). Furthermore, the extracts of the plant exhibited significant antifungal, antibacterial, antimicrobial, anticancer, anti-inflammatory, antidiabetic, antithrombotic and antitumor properties that could be further exploited by the pharmaceutical industry and contribute to the promotion of human health (Elberry et al., 2014; Petropoulos et al., 2017; Shabir et al., 2022). Recent reports have also highlighted the significant antioxidant properties of onion, which are responsible for neutralizing free radicals and further preventing diseases associated with oxidative damage, such as cardiovascular disease and various types of cancer (Cioni et al., 2025).

The distinct aroma and flavor of onion is associated with the rupture of plant tissues after either cutting or breakage during processing which results in the hydrolyzation of sulfur-containing compounds such as S-alk(en)yl-l-cysteine sulfoxides (ACSOs) by alliinase in order to produce ammonia, sulfenic acids and pyruvic acid as well as a mixture of both volatile and nonvolatile sulfur compounds (Liguori et al., 2017; Petropoulos et al., 2017). Moreover, the concentration of pyruvic acid detected in onion tissues after hydrolysis reaction takes places is an indicator of pungency with significant variation among different cultivars and/or landraces (Yoo et al., 2006; Liguori et al., 2017; Loredana et al., 2019).

Onion bulbs are a rich source of phytochemicals with a great variation within the vast genetic pool of the species, including a high content of phenolic compounds and specifically flavonoids (e.g. quercetin and its derivatives) but also vitamins and minerals (Chakraborty et al., 2022; Kumar et al., 2022). Phenolic composition and flavonoids content in particular is also associated with skin and flesh color of bulbs with two subgroups of compounds being detected, namely anthocyanins which are commonly found in onion genotypes with red/purple color and flavanols which impart the yellow and brown color of bulb skin (Piechowiak et al., 2020; Cozzolino et al., 2021; Brahimi et al., 2022; Gorrepati et al., 2024). Moreover, quercetin has been suggested to have a key role in the antioxidant properties of onion through the scavenging of free radicals, the reduction of oxidative stress and the support of enzymatic defensive systems (Sagar et al., 2022; Imran et al., 2025). Other significant antioxidant constituents include sulfur compounds such as di- and trisulfides, while allicin is highly linked with the antimicrobial potential of the species. For example, extracts from either fresh flowers or leaves demonstrated significant antibacterial, antifungal and antimicrobial activities by exhibiting inhibitory effects towards Gram-positive (*Staphylococcus aureus*) and Gram-negative bacteria (*Escherichia coli*, *Salmonella* spp. and *Klebsiella* spp., *Pseudomonas aeruginosa* and *Helicobacter pylori*) (Gabriel et al., 2022; Barbu et al., 2023). However, these bioactive properties display a wide range of variability due to large genetic diversity of onion, the extraction protocol, the storage conditions, the plant and processing technologies (Kim et al., 2024).

Throughout the cultivation history of onion over the years, there has been detected a broad variability mostly in terms of morphological traits such as the shape, the size and the color of the bulb due to differences in genetic material and the acclimatization and farmer’s selection process under variable edaphoclimatic conditions. However, although distinct ecotypes and local landraces present a vast genetic pool the current trends for increased yield that the agricultural sector has to follow has marginalized this valuable material in favor of modern and more productive cultivars and hybrids. The variable edaphoclimatic conditions across Greece, facilitate the existence of several onion landraces with unique characteristics in terms of quality traits and adaptability to specific conditions that are still underexplored. Therefore, in this work we assessed for the first time the phytochemical properties of four Greek onion local landraces that are locally cultivated aiming to highlight their nutritional value, metabolic profile and bioactivities and identify elite and high-quality genetic material that could be reintegrated into breeding programs and valorized in the nutraceutical sector based on their phytochemical and metabolic map and their functional properties.

## 2 Materials and methods

### 2.1 Sampling methodology

**Figure 1:**
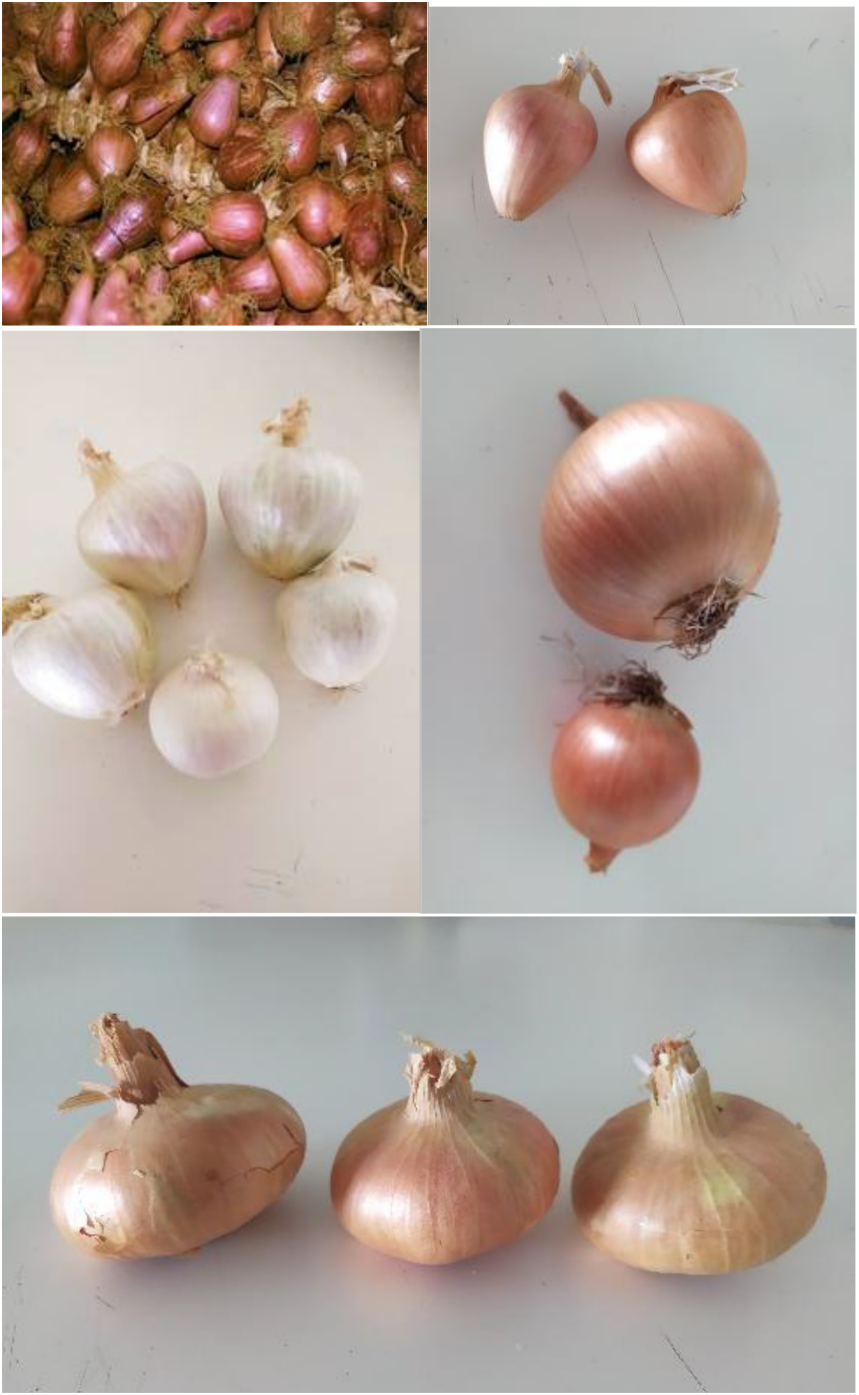
Dried onion bulbs of the studied onion genotypes (ON1 to ON5: from upper left to bottom). Credits: personal record of Prof. Spyridon A. Petropoulos.

Samples of onion bulbs (10 kg of dried onion bulbs) were sampled from bulk lots from commercial farms in July 2023 at marketable maturity and after curing for 2 weeks from farmers located in the region of Thiva (3 local landraces and one commercial cultivar; 38°16’54.1”N, 23°06’36.8”E) and Evoia (one local landrace; 38°31’26.9”N, 24°09’57.5”E). The plants were cultivated according to best practice guides followed in each region. The description of the studied genotypes is cited in **Table 1**, while **Figure 1** depicts the bulbs of each genotype. Prior to processing, bulbs were cleaned with distilled water, wiped with absorbed paper and then let to dry under dark conditions at room temperature. After drying, bulbs were peeled (tunic removal) and the flesh was cut in cubes (approximately 0.5 cm wide) and stored at deep-freezing conditions (-80°C) in air sealed bags. Then, onion samples were lyophilized and ground in fine powder (≈20 mesh) with a mortar and pestle and stored under deep-freezing conditions (-80°C) until further analysis.

**Table 1:** Description of the studied onion samples.

Code	Description
ON1	Onion landrace from the region of Octonia (oblong shape, red skin with red to purple flesh)
ON2	Onion landrace from Thiva region (rhomboid shape, pale red skin with white flesh)
ON3	Onion landrace from Thiva region (globular shape, white skin with white to red flesh (the outer scales))
ON4	Commercial cultivar (cv. Mirsini; SeedTech, Hellas) de Grano type (global shape, pale red skin with white flesh)
ON5	Onion landrace from Thiva region (elliptical shape, pale red skin with white flesh)

### 2.2 Preparation of hydroethanolic extract

Extracts were obtained through maceration from freeze-dried samples by mixing 3 g of each sample with 90 mL of an 80% ethanol solution. Following agitation for 1 hour at ambient temperature, the suspensions were filtered using paper filters. The residues underwent a second extraction under the same conditions. The hydroethanolic extracts from both rounds were merged and then followed the evaporation of ethanol at 40°C under vacuum. The resultant aqueous fractions were subsequently frozen and lyophilized to obtain dried extracts that were subsequently employed for evaluating the biological properties of the samples and their phenolic compound profiles.

### 2.3 Nutritional composition

The nutritional profile of the samples, e.g. lipid, protein, ash, total dietary fiber, carbohydrate content, and total energy value, was determined following the procedures described by the Association of Official Analytical Chemists (AOAC; (AOAC, 2019). Lipid extraction was carried out in a Soxhlet apparatus for 7 hours, using petroleum ether as solvent; the amount of protein was measured using the macro-Kjeldahl method, involving acid digestion, distillation, and titration steps, applying a conversion factor of 6.25; ash content was obtained after the incineration of samples at 550 ± 10°C for 7 hours using a muffle furnace; total dietary fiber (TDF) was determined by an enzymatic–gravimetric method (TDF-100A Kit; Sigma Aldrich, St. Louis, MO, USA); and the content of carbohydrates was calculated by difference. All the results were presented as g per 100 g of dry weight (dw). The energy value was estimated using the equation below:


Energy (kcal/100g dw) = 4 × (g protein + g carbohydrate) + 9 × (g fat)

### 2.4 Chemical profile of hydrophilic compounds

#### 2.4.1 Free sugars

Free sugars were quantified following an ethanolic extraction procedure. Briefly, each dried and powdered sample (1 g) was mixed with 40 mL of 80% ethanol and 1 mL of melezitose solution (25 mg/mL), used as the internal standard (IS). The mixture was maintained in a water bath at 80°C for 90 min under constant agitation. After extraction, the suspensions were filtered through paper filters, and then followed the evaporation of solvent at 40°C. The resulting aqueous phase was brought to a final volume of 5 mL with distilled water, filtered via 0.2 µm nylon filters, and transferred to vials for chromatographic analysis. The determination was carried out using an HPLC system, as described by Spréa et al. (2020), while the chromatographic conditions and the identification of compound were described by the same authors (Spréa et al., 2020). The results were presented as g per 100 g of dw.

#### 2.4.2 Organic acids

Organic acids were extracted and assessed as described by Barros et al. (2013b), while the quantification was performed by Ultra-Fast Liquid Chromatography (UFLC) using the conditions described by the same authors. The results were presented as g per 100 g of dw.

### 2.5 Chemical profile of lipophilic compounds

#### 2.5.1 Fatty acids

The Fatty Acid Methyl Ester (FAME) composition was analyzed using the lipid fraction obtained from the crude fat determination, as described in section 2.3, following the protocol of Obodai et al. (2017). The resulting FAMEs were analyzed by Gas Chromatography (GC) following the same protocol, while the acquisition and processing of data were carried out with Clarity 4.0.1.7 software (DataApex, Prague, Czech Republic). The composition of fatty acids was presented as the relative percentage (%) of each identified compound.

#### 2.5.2 Tocopherols

Tocopherols extraction was performed according to the methodology reported by Spréa et al. (2020), using a chromatographic system and conditions already described by the same authors. Data acquisition and integration were performed with Clarity 2.4 software (DataApex, Prague, Czech Republic). The quantification of compounds was carried according to the fluorescence response of each compound, using the internal standard (IS) method, as well as the comparison with analytical standards. The results were presented as mg per 100 g of dw.

### 2.6 Identification and quantification of phenolic compounds

The phenolic composition of the hydroethanolic extracts from the maceration was analyzed by UPLC coupled to a DAD, following the protocol and the conditions previously reported by Bessada et al. (2016). The acquisition and processing of data were performed using Xcalibur^®^ software (version 2.2.0.48; Thermo Fisher Scientific). Identification of phenolic compounds was performed using the comparison of their UV–vis spectra, retention times, and mass fragmentation patterns with reference standards and literature data when available. Quantification was achieved by external calibration using commercial phenolic standards, with detection at their maximum absorbance wavelengths. When reference standards were not available, the compounds were quantified relatively to other molecules bearing the same phenolic group. The results were presented as milligrams per gram of extract (mg/g extract).

### 2.7 Bioactive properties

#### 2.7.1 Antioxidant activity

The antioxidant capacity of the hydroalcoholic extracts was determined *in vitro* using the Thiobarbituric Acid Reactive Substances (TBARS) assay according to the methodology of Sarmento et al. (2015). The inhibition percentage was estimated using the equation: Inhibition (%) = [(A − B)/A] × 100, where A and B represent the absorbance of control treatment and the analyzed sample, respectively. The EC_50_ value (extract concentration that achieves 50% inhibition, mg/mL) was determined by plotting the inhibition percentage towards extract concentration. Trolox was implemented as a reference antioxidant standard.

#### 2.7.2 Antibacterial activity

The antibacterial activity was assessed according to the rapid colorimetric method as reported by Pires et al. (2018). The tested gram-positive bacteria were: *Staphylococcus aureus* (ATCC 25923), *Bacillus cereus* (ATCC 11778) and *Listeria monocytogenes* (ATCC 19111), while the gram-negative bacteria were: *Escherichia coli* (ATCC 25922), *Salmonella enterocolitica* (ATCC 13076), *Pseudomonas aeruginosa* (ATCC 9027), *Yersinia enterocolitica* (ATCC 8610) and *Enterobacter cloacae* (ATCC 49741). All the tested bacteria were food isolates (Frilabo, Porto, Portugal). Two negative controls were implemented, one with TSB and another one with the extract. Two positive controls were used, e.g. TSB and each inoculum and one more with medium, antibiotics, and bacteria. Ampicillin and streptomycin were used for all the bacteria tested and methicilin was used additionally for *S. aureus*. The minimum inhibitory concentration (MIC) represents the lowest concentration that inhibits the visible bacterial growth, based on the change of color from yellow to pink in the case that the microorganisms were viable, while the minimum bactericidal concentration (MBC) was assessed as the lowest concentration to yield no growth of the studied bacteria.

#### 2.7.3 Antifungal activity

Antifungal activity was determined using the procedure described by Heleno et al. (2013) using *Aspergillus fumigatus* (ATCC 204305) and *A. brasiliensis* (ATCC 16404), as well as ketoconazole (Frilabo, Porto, Portugal) as positive control. MIC determination was defined as the lowest concentrations without visible growth of fungi, while minimum fungicidal concentration (MFC) was the lowest concentration with no visible growth of the studied fungi (e.g. 99.5% killing of the original inoculum). Ketoconazole (Frilabo, Porto, Portugal) was implemented as positive control.

### 2.8 Statistical analysis

All the assays were carried out in triplicate using three samples (n=3). The data were presented as mean values with standard deviations (SD). The analysis of results was performed with one-way analysis of variance (ANOVA), while the comparison of means was performed with Duncan’s Multiple Range Test (DMRT) at p = 0.05.

## 3 Results and discussion

### 3.1 Chemical composition

The findings of the current study regarding the nutritive value and free sugars and organic acids composition are cited in **Table 2**. Based on these results, a significant variability in nutritional value was observed with differences between both the genotypes of the same region as well as between genotypes from different regions. In particular, fat, proteins, ash and carbohydrates content ranged between 0.36 and 1.09 g/100 g dw, 5.06 and 14.50 g/100 g dw, 3.33 and 4.51 g/100 g dw and 68.1 and 73.9 g/100 g dw, while energetic value ranged between 355.0 and 366.8 Kcal/100 g dw. Moreover, ON2 samples recorded the highest fat and proteins content and the highest energetic value, while ON1 and ON4 samples had the highest carbohydrates and ash content, respectively.

**Table 2:** Nutritional, energetic value and hydrophilic compounds content of the studied onion samples (mean ± SD, *n=3*).

	ON1	ON2	ON3	ON4	ON5
Nutritional value	(g/100 g dw)				
Fat	1.05 ± 0.02^a^	1.09 ± 0.05^a^	0.86 ± 0.03^b^	0.36 ± 0.08^d^	0.79 ± 0.02^c^
Proteins	5.06 ± 0.09^e^	14.50 ± 0.01^a^	9.89 ± 0.01^c^	9.84 ± 0.06^d^	10.10 ± 0.04^b^
Ash	3.91 ± 0.01^b^	3.75 ± 0.01^d^	3.78 ± 0.04^c^	4.51 ± 0.01^a^	3.33 ± 0.01^e^
Total Dietary Fibers (%)	13.78 ± 0.03^b^	12.6 ± 0.2^d^	12.9 ± 0.1^c^	14.6 ± 0.8^a^	11.9 ± 0.5^e^
Carbohydrates	76.2 ± 0.1^a^	68.1 ± 0.2^e^	72.50 ± 0.06^c^	70.7 ± 0.5^d^	73.9 ± 0.3^b^
Energy (Kcal/100 g dw)	362.07 ± 0.03^b^	365.3 ± 0.1^a^	363.3 ± 0.1^c^	355 ± 1^d^	366.8 ± 0.6^a^
Hydrophilic compounds					
Free sugars	(g/100 g dw)				
Fructose	5.49 ± 0.07^b^	2.70 ± 0.06^d^	2.60 ± 0.03^e^	18.88 ± 0.05^a^	3.30 ± 0.05^c^
Glucose	6.04 ± 0.07^b^	4.86 ± 0.04^c^	4.36 ± 0.03^d^	20.1 ± 0.1^a^	2.57 ± 0.03^e^
Sucrose	9.43 ± 0.06^a^	7.49 ± 0.06^c^	4.81 ± 0.01^d^	8.52 ± 0.08^b^	4.74 ± 0.03^e^
Trehalose	0.20 ± 0.02^c^	0.20 ± 0.01^c^	0.23 ± 0.02^b^	0.36 ± 0.03^a^	0.21 ± 0.01^c^
Unknown compounds	3.70 ± 0.04^a^	3.41 ± 0.03^b^	2.90 ± 0.03^c^	1.20 ± 0.01^e^	2.54 ± 0.05^d^
Total	24.9 ± 0.3^b^	18.66 ± 0.02^c^	14.89 ± 0.01^d^	49.1 ± 0.1^a^	13.4 ± 0.2^e^
Organic acids	(g/100 g dw)				
Oxalic acid	0.45 ± 0.04^d^	0.55 ± 0.01^c^	0.61 ± 0.04^b^	1.27 ± 0.01^a^	0.62 ± 0.01^b^
Pyruvic acid	0.18 ± 0.01^b^	0.15 ± 0.01^d^	0.14 ± 0.01^e^	0.23 ± 0.01^a^	0.17 ± 0.01^c^
Citric acid	3.3 ± 0.2^a^	3.1 ± 0.1^b^	2.86 ± 0.07^c^	3.1 ± 0.3^b^	3.1 ± 0.1^b^
Succinic acid	1.6 ± 0.1^d^	3.0 ± 0.1^b^	3.00 ± 0.09^b^	7.5 ± 0.4^a^	2.4 ± 0.1^c^
Fumaric acid	tr	tr	tr	tr	tr
Total	5.5 ± 0.2^e^	6.75 ± 0.01^b^	6.60 ± 0.02^c^	12.1 ± 0.2^a^	6.2 ± 0.2^d^

Means in the same line followed by different line letters are significantly different according to Duncan’s Multiple Range test (DMRT) at p<0.05.

tr, traces.

These results agree with those of Petropoulos et al. (2015) who suggested a significant variation in the nutritional profile of different onion genotypes, including one local landrace and three commercial cultivars, while similarly to our study the studied local landrace recorded a better nutritive value in comparison to commercial hybrids and cultivars. The superiority of landraces over the commercial cultivars could be attributed to genotypic differences (Ariyama et al., 2006; Liguori et al., 2017; Loredana et al., 2019; Cozzolino et al., 2021), as well as to adaptation growing conditions since landraces are expected perform better under specific edaphoclimatic conditions in the designated growing areas due to continuous selection from farmers over the years (Monteverde et al., 2015; Brahimi et al., 2022; Ochar and Kim, 2023). Similarly, Shukla et al. (2024) documented that the content of onion bulbs cultivated at high altitudes was larger in terms of crude protein, crude fat and total carbohydrates in comparison to the same genotype cultivated at low altitudes suggesting that environmental conditions may regulate the chemical composition of the species. On the other hand, Diriba-Shiferaw et al. (2014) reported that the application of different nitrogen, phosphorus and sulfur rates and the soil type may affect the nutritional profile of garlic bulbs, especially in regards to protein content. Moreover, significant differences were detected between the genotypes coming from the same region namely ON2, ON3, ON4 and ON5, thus indicating that apart from agronomic practices and environmental conditions the genotype may also play a crucial role to nutritional profile of onion bulbs (Petropoulos et al., 2018).

A significant variation was also recorded in relation to total dietary fibers where the commercial cultivar (ON4) has the highest overall content (14.6%), sample ON5 showed the lowest concentration (11.9%; **Table 2**). The results of this study are in alignment with those of Benítez et al. (2011) who suggested significant differences in total dietary fibers content between two onion cultivars and recorded a higher content of total fibers in whole onion bulbs compared to our study, whereas the content of inner scales was in the same range as in our study since the larger part of dietary fibers was present in usually discarded bulb tissues (e.g. top and bottom, outer scales and skin). The genotypic variation of total fiber content in onion bulbs was also confirmed by Rodríguez Galdón et al. (2009) who tested six cultivars originated from Spain.

The free sugars composition of the studied onion samples is cited in **Table 2**, where glucose was the most abundant compound (2.57 to 20.1 g/100 g dw) followed by fructose (2.60 to 18.88 g/100 g dw), sucrose (4.74 to 8.52 g/100 g dw) and trehalose (0.20 to 0.36 g/100 g dw), while an unidentified compound was also detected in amounts that ranged from 1.20 to 3.70 g/100 g dw. A significant variation was recorded among the onion genotypes, with the commercial cultivar (ON4) having the highest content of individual free sugars (except for the unidentified compound whose content was the lowest in this particular sample), thus also presenting the largest amount of total free sugars (49.1 g/100 g dw). Similarly to our work, Metrani et al. (2020) also reported that glucose, fructose and sucrose were the main detected sugars in the bulbs of two red onion cultivars; however, they suggested that the content of individual free sugars varied according to the genotype. Similar trends were also recorded by Major et al. (2023) who reported that glucose was the most abundant free sugar in a sweet onion landrace (e.g. Premanturska kapula) followed by fructose and sucrose, while according to their findings the bulb size may affect individual free sugars composition with smaller bulbs (approximately 200 g) having higher amounts of glucose and larger ones (approximately 470 g) a higher content of sucrose. The differences among genotypes in terms of free sugars composition was also highlighted by Arena et al. (2024), although they recorded either glucose or fructose as the major compounds. Other studies also point out that free sugars composition in onion bulbs may be affected not only by the genotype, but also from the environmental conditions during the growing period, the growth stage of the plant at harvest and the storage conditions (Benkeblia et al., 2005; Yoo et al., 2006; Rodríguez Galdón et al., 2009). Considering that free sugars and particularly fructose and glucose content is associated with bulb quality and likeability and sweetness of onion bulbs (Magwaza and Opara, 2015), the genotypic variation of free sugars composition could be valorized by producing bulbs from specific genotypes based on consumers preferences.

The organic acids profile is cited in **Table 2**. The main identified compound was succinic acid (1.6-7.5 g/100 g dw), followed by citric, oxalic and pyruvic acid (2.86-3.3 g/100 g dw, 0.45-1.27 g/100 g dw and 0.14-0.23 g/100 g dw, respectively), while traces of fumaric acid were also detected. The commercial cultivar (sample ON4) had the largest content of total and individual organic acids, expect for citric acid where sample ON1 recorded the highest content. In contrast, the lowest content of oxalic acid was measured in sample ON1 which also presented the lowest content of succinic acid, while the lowest amounts of pyruvic acid were identified in sample ON3. According to Colina-Coca et al. (2014) who assessed the nutritional composition of processed onion, the same organic acids were also detected in bulb tissues, while the same authors detected malic acid, formic acid and acetic acid, a difference that could be attributed to the processing treatment, the genotype and the analytical protocol. In contrast, Liguori et al. (2017) suggested citric and malic acid as the major organic acids in five Italian onion landraces, while the same major organic acids were suggested by Petropoulos et al. (2015) in various onion genotypes cultivated in Greece. However, in the study of Rodríguez Galdón et al. (2008a) a different organic acids profile was recorded in six onion cultivars cultivated in Spain with glutamic acid being identified as the major compounds, while Vargas et al. (2010) observed a significant interaction between environment and genotype in the expression of pyruvic acid in garlic. Moreover, agronomic practices such as transplanting time and fertilization regime may also alter the profile of organic acids in onion bulbs (Caruso et al., 2014; Pérez-Gregorio et al., 2014). Therefore, our findings and those obtained from other literature reports suggest that the genotype, the growing conditions and the agronomic practices applied during cultivation may significantly affect not only the organic acids profile of onion bulbs but also the content of individual compounds.

The lipophilic compounds of the onion samples were further examined regarding the chemical composition of fatty acids and tocopherols, while the obtained hydroethanolic extracts were subjected to additional analysis to estimate their antioxidant activity, as cited in **Table 3**. The main fatty acids identified in all the samples were linoleic (C18:2n6c; 40.8-47.1%) and oleic acid (C18:1n9c; 14.3-17.7%), followed by palmitic (C16:0; 8.47-11.9%), eicosenoic (C20:1; 6.7-10.1%), and behenic acid (C22:0; 4.4-5.27%). A similar profile of the major fatty acids was reported by Petropoulos et al. (2015) who also identified linoleic and oleic acid in high amounts in four onion genotypes; however, the same authors suggested a higher content of palmitic and stearic acid in amounts that varied among the studied samples, as well as lesser amounts of eicosenoic and behenic acid. Similar findings were recorded in the report of Farag et al. (2017) where linoleic, palmitic, oleic and stearic acid were the most abundant compounds, whereas Tsiaganis et al. (2006) detected high amounts of linoleic, palmitic and oleic acid in amounts that varied among different onion samples obtained from a local market. In contrast, Bello et al. (2013) suggested palmitic and oleic acids as the major compounds in whole onion bulbs or in top-bottom waste, while linoleic acid was the richest compound in the outer scales. Therefore, onion bulbs showed a varied fatty acids profile in literature reports depending on the genotype, while processing treatments, the part of onion bulb and the origin of samples may also affect the individual fatty acids content (Bello et al., 2013; Petropoulos et al., 2015).

**Table 3:** Chemical composition of lipophilic compounds in the studied onion samples and antioxidant activity of their hydroethanolic extracts (mean ± SD, n=3).

Fatty acids	ON1	ON2	ON3	ON4	ON5
	Relative percentage (%)				
C12:0	0.156 ± 0.002^e^	0.18 ± 0.004^d^	0.193 ± 0.004^c^	0.361 ± 0.001^a^	0.22 ± 0.01^b^
C13:0	0.21 ± 0.01	nd	nd	nd	nd
C14:0	0.263 ± 0.004^d^	0.27 ± 0.03^d^	0.293 ± 0.005^c^	0.459 ± 0.02^a^	0.35 ± 0.02^b^
C15:0	1.066 ± 0.001d	1.06 ± 0.04^d^	1.37 ± 0.04^a^	1.12 ± 0.04^c^	1.33 ± 0.02^b^
C16:0	8.47 ± 0.06^e^	10.0 ± 0.4^c^	10.4 ± 0.2^b^	11.9 ± 0.1^a^	9.3 ± 0.2^d^
C16:1	2.4 ± 0.1^c^	2.378 ± 0.008^c^	2.81 ± 0.05^b^	4.11 ± 0.1^a^	2.27 ± 0.01^d^
C17:0	0.78 ± 0.06^c^	0.99 ± 0.08^a^	1.00 ± 0.02^a^	0.89 ± 0.02^b^	0.94 ± 0.02^ab^
C18:0	2.1 ± 0.1^d^	2.4 ± 0.2^c^	2.3 ± 0.1^c^	3.38 ± 0.1^a^	2.66 ± 0.08^b^
C18:1n9c	15.1 ± 0.2^d^	17.2 ± 0.2^b^	14.3 ± 0.1^e^	17.7 ± 0.3^a^	15.5 ± 0.1^c^
C18:2n6c	44.7 ± 0.2^b^	44.2 ± 0.02^c^	47.1 ± 0.4^a^	40.8 ± 0.3^d^	45.0 ± 0.3^b^
C18:3n3	3.4 ± 0.3^a^	2.35 ± 0.04^b^	2.37 ± 0.1^b^	2.41 ± 0.1^b^	2.1 ± 0.05^c^
C20:0	1.17 ± 0.05^d^	1.68 ± 0.05^c^	2.2 ± 0.1^a^	1.21 ± 0.02^d^	1.94 ± 0.07^b^
C20:1	9.02 ± 0.3^b^	10.1 ± 0.2^a^	8.6 ± 0.2^c^	6.7 ± 0.1^d^	10.1 ± 0.4^a^
C20:2	1.02 ± 0.04	n	nd	nd	nd
C21:0	0.52 ± 0.03	nd	nd	nd	nd
C22:0	4.4 ± 0.2^c^	4.95 ± 0.04^b^	4.52 ± 0.04^c^	5.27 ± 0.1^a^	5.18 ± 0.07^a^
C22:1	1.28 ± 0.08	nd	nd	nd	nd
C23:0	1.11 ± 0.03^b^	0.74 ± 0.01^e^	0.84 ± 0.03^d^	1.32 ± 0.04^a^	0.905 ± 0.001^c^
C24:0	2.35 ± 0.02^a^	1.50 ± 0.02^d^	1.73 ± 0.01^c^	2.41 ± 0.08^a^	2.23 ± 0.09^b^
C24:1	0.57 ± 0.01	nd	nd	nd	nd
SFA	22.6 ± 0.3^e^	23.8 ± 0.1^d^	24.8 ± 0.3^c^	28.29 ± 0.02^a^	25.04 ± 0.09^b^
MUFA	28.3 ± 0.1^c^	29.7 ± 0.1^a^	25.7 ± 0.2^e^	28.5 ± 0.5^b^	27.9 ± 0.3^d^
PUFA	48.1 ± 0.5^b^	46.5 ± 0.1^d^	49.4 ± 0.5^a^	43.2 ± 0.4^e^	47.1 ± 0.4^c^
Tocopherols	(mg/100 g dw)				
*α*-Tocopherol	0.082 ± 0.001^d^	0.149 ± 0.004^a^	0.102 ± 0.001^b^	0.104 ± 0.001^b^	0.087 ± 0.08^c^
Total	0.082 ± 0.001^d^	0.149 ± 0.004^a^	0.102 ± 0.001^b^	0.104 ± 0.001^b^	0.087 ± 0.08^c^

Means in the same line followed by different line letters are significantly different according to Duncan’s Multiple Range test (DMRT) at p<0.05.

SFA, saturated fatty acids; MUFA, monounsaturated fatty acids; PUFA, polyunsaturated fatty acids, Trolox: 5.4 ± 0.3 (mg/mL).

Polyunsaturated fatty acids (PUFA; 43.2-49.4%) was the major class of fatty acids, followed by MUFA (monounsaturated fatty acids; 25.7-29.7%) and SFA (saturated fatty acids; 22.6-28.29%) (**Table 3**). Moreover, significant differences among the studied samples were recorded with samples ON2 and ON3 showing the largest content of MUFA and PUFA, respectively, while the commercial cultivar (ON4) was the richest in SFA. Our findings are in line with those of Petropoulos et al. (2015) who suggested that in another Greek onion landrace (e.g. “Vatikiotiko”) PUFA was the most abundant class, while MUFA and SFA were detected in similar amounts. Moreover, Rizwani and Shareef (2011) suggested that the content of PUFA was more than double of SFA and significantly higher than MUFA in raw onion bulbs. Polyunsaturated fatty acids were also the most abundant class of fatty acids in onion seeds (Parry et al., 2006; Amalfitano et al., 2019), while Bello et al. (2013) suggested that the fatty acids profile may differ depending on the bulb part (e.g. whole bulb, top-bottom and outer scale). Moreover, the PUFA/SFA ratio was higher than 0.45 for all the studied onions (1.52-2.13), although the local landraces recorded higher values compared to the commercial cultivar (**Table 3**). This finding shows the high nutritional value of onion bulbs, since high values for the PUFA/SFA ratio are associated with prevention of cardiovascular diseases and atherosclerosis (Diriba-Shiferaw et al., 2014; Yalcin and Kavuncuoglu, 2014).

The composition of tocopherols is cited in **Table 3**, where α-tocopherol was the only compound detected in varied amounts among the studied samples (0.082-0.149 mg/100 g dw), while the largest and smallest overall content was measured in samples ON2 and ON1, respectively. Our findings are in accordance with those suggested by Chun et al. (2006) who detected only α-tocopherol and α-tocotrienol in raw white onion bulbs, while Petropoulos et al. (2015) also detected the same compound in the bulbs of three commercial cultivars and one local landrace cultivated in Greece. The same isoform of vitamin E was also the most prevalent compound in onion seed oils, while the rest of the isoforms were identified in lesser amounts (Parry et al., 2006; Topkafa, 2016; Matthäus et al., 2021). The profile and content of tocols (e.g. tocopherols and tocotrienols) are crucial for the antioxidant mechanism of plants, while they are associated with lipid peroxidation inhibition and the prevention of human chronic diseases (Tiwari and Cummins, 2009; Petropoulos et al., 2019). Therefore, the elucidation of the pre- and postharvest conditions that may influence the biosynthesis of these compounds will significantly increase the quality and functionality of vegetable products such as onion bulbs.

### 3.2 Phenolic compounds

**Figure 2:**
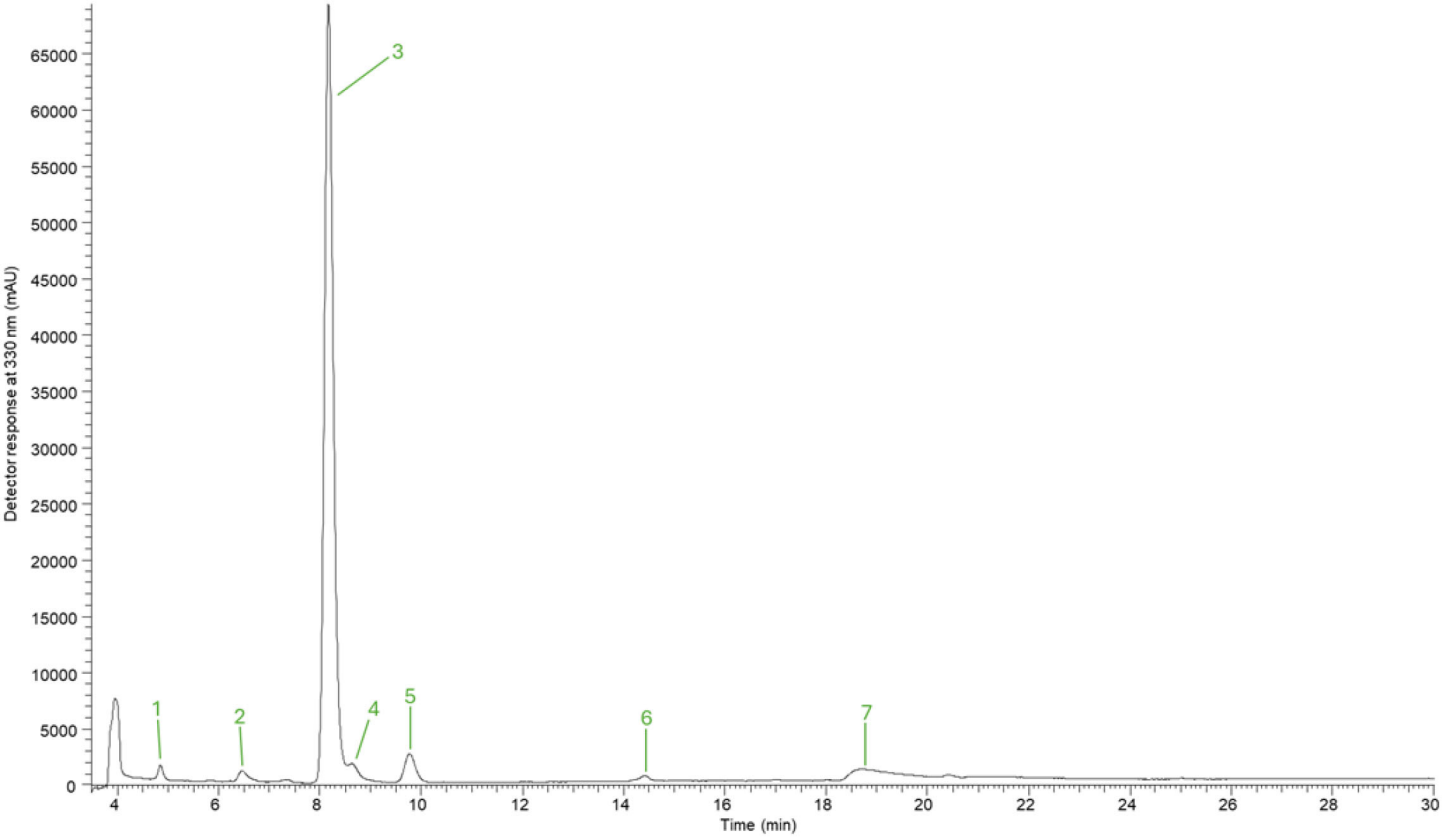
Profile of phenolic compounds in the hydroethanolic extracts obtained from ON1 sample. The peaks are described in **Table 1**.

The composition of polyphenols in the hydroethanolic onions extracts, as well as the respective retention time (Rt), wavelengths of maximum absorption in the visible region (λ_max_), mass spectra data are described in **Table 4**, while **Figure 2** depicts a chromatograph for one of the studied samples (ON1). Seven compounds were detected, including five flavonoids and two phenolic acids. In general, flavonoids were the major class of polyphenols comprising 96-99% of total phenolic compounds in most of the samples, except for ON3 sample where only one flavonoid and one phenolic acid were detected with the latter being the most abundant one. Regarding the detected flavonoids, quercetin-*O*-hexoside-*O*-hexoside (peak 3) was the most abundant phenolic compound in all the samples with a content that varied greatly from 0.02 mg/g extract to 3.15 mg/g extract (samples ON3 and ON5, respectively). Other significant compounds were quercetin-*O*-hexoside (peak 7) with the largest content being detected in ON5 sample (0.64 mg/g extract), as well as kaempferol-*O*-dihexoside (peak 4; the highest content was recorded in ON2 sample; 0.18 mg/g extract). Caffeoyl-N-Tryptophan (peak 2) was detected in amounts that varied between 0.05 mg/g extract (ON1) and 0.14 mg/g extract (ON4), while only traces of sinapic acid hexoside (peak 1) were found in all the samples. The content of phenolic compounds recorded a great variation among the tested genotypes, with ON5 having the highest content for all the detected compounds, except for caffeoyl-N-Tryptophan where the largest content was recorded in ON4 sample.

**Table 4:** Retention time (Rt), wavelengths of maximum absorption in the visible region (Λmáx), mass spectra data, tentative identification, and quantification of the phenolic compounds found in hydroethanolic onions extracts (*n* = 3, mean ± SD).

Peak n.°	Rt (min)	λmax (nm)	[M – H]^–^ (m/z)	MS^2^ fragments (m/z, %)	Assignment	Quantification (mg/g of extract)				
						ON1	ON2	ON3	ON4	ON5
1	4.85	298	385	223 (100), 164 (82), 179 (62), 208 (51), 149 (12)	Sinapic acid hexoside (1)	tr	tr	tr	tr	tr
2	6.46	328	365	203 (100)	Caffeoyl-*N*-Tryptophan (2)	0.05 ± 0.01^d^	0.07 ± 0.002^b^	0.060 ± 0.001^c^	0.140 ± 0.006^a^	0.07 ± 0.01^b^
3	8.17	265, 344	625	301 (100), 300 (51), 463.0882 (50)	Quercetin-*O*-hexoside-*O*-hexoside (3–6)	3.00 ± 0.02^b^	2.56 ± 0.01^d^	0.02 ± 0.01^e^	2.68 ± 0.03^c^	3.15 ± 0.04^a^
4	8.62	266, 345	609	609 (100), 446 (76), 283 (64), 285 (26), 447 (20), 489 (4)	Kaempferol-*O*-dihexoside (7)	0.160 ± 0.004^b^	0.180 ± 0.006^a^	tr	0.14 ± 0.02^c^	0.16 ± 0.02^b^
5	9.77	266, 345	639	313 (100), 476 (51), 315 (19), 477 (17), 314 (9), 519 (3)	Isorhamnetin-*O*-dihexoside (2,8)	0.14 ± 0.01^d^	0.160 ± 0.008^b^	nd	0.150 ± 0.005^c^	0.210 ± 0.001^a^
6	14.4	–	463	300 (100), 301 (42)	Quercetin-3-*O*-glucoside ^(Std)^	0.040 ± 0.008^c^	0.040 ± 0.001^c^	nd	0.050 ± 0.002^b^	0.070 ± 0.007^a^
7	19.10	–	463	301 (100), 300 (6)	Quercetin-*O*-hexoside (3–5,9)	0.47 ± 0.01^c^	0.50 ± 0.02^b^	nd	0.57 ± 0.06^a,b^	0.64 ± 0.06^a^
					Total phenolic acids	0.05 ± 0.01^d^	0.070 ± 0.002^b^	0.060 ± 0.001^c^	0.140 ± 0.006^a^	0.070 ± 0.001^b^
					Total flavonoids	3.81 ± 0.02^b,c^	3.430 ± 0.001^d^	0.02 ± 0.01^e^	3.590 ± 0.007^c,d^	4.24 ± 0.04^a^
					Total phenolic compounds	3.86 ± 0.02^b^	3.500 ± 0.003^c,d^	0.08 ± 0.01^e^	3.73 ± 0.01^b,c^	4.30 ± 0.04^a^

tr, traces; nd, not detected.

Statistically significant differences (*p* < 0.05) between samples were determined by a one-way ANOVA, using Tukey’s significant difference (HSD), and are indicated by different Latin letters. (1- Hernández et al. (2021); 2- Barros et al. (2012); 3- Ullah et al. (2022); 4- Nile et al. (2021); 5- Farag et al. (2017); 6- (Barros et al., 2013a); 7- Spínola et al. (2016); 8- Cozzolino et al. (2021); 9- Joković et al. (2024); Std- standard).

Similarly to our work, Cozzolino et al. (2021) suggested that quercetin glycosides were the major compounds in the bulbs from various onion landraces with coppery outer color, while flavonoids and quercetin derivatives were the major class of phenolic compounds in two sweet red onions cultivated in Italy (Metrani et al., 2020). Moreover, Rodríguez Galdón et al. (2008b) also suggested that quercetin was the main flavonoid in six onion cultivars cultivated in Tenerife (including five local landraces from this region); however, the fact that total flavonoids comprised only 14.9-22.2% of total phenolic compounds indicates that other phenolic compounds were also present but not identified in that study. According to the work of Petropoulos et al. (2015), phenolic acids were the most prevalent class of polyphenols in four onion landraces cultivated in Greece, a result that could be assigned to genotype and growing conditions specificities, as well as to differences in the color of flesh. Similarly, Loredana et al. (2019) detected seven phenolic acids and four flavonoids in the bulbs of four Italian onion landraces; however, the same authors suggested a variable profile among the tested genotypes, with quercetin being the major polyphenol.

The color of bulbs is commonly related with the content and composition of polyphenols with genotypes with yellow to cream color bulbs showing higher content of flavonoids, while red onions are richer in phenolic acids and anthocyanins (Kim et al., 2024). However, the reports regarding effect of the bulb skin color on the profile of phenolic compounds are not consistent since there are studies which detected flavonoids as the major compounds in red onions too (Metrani et al., 2020), while a great variation in the content of total phenolic compounds and total flavonoids has been recorded depending on the genotype (Cioni et al., 2025). Therefore, apart from the skin color the part of the bulb and the color of flesh are also essential for polyphenols composition with outer scales showing a varied content of phenolic acids and flavonoids compared to inner ones (Benmalek et al., 2013; Cheng et al., 2013), while bulb size may also have an impact on flavonoids profile and the total phenolic compounds content (Major et al., 2023). Additionally, the contradictory results in literature reports could be associated with different extraction protocols, the sample preparation (fresh or dried bulbs and discard of outer scales), and the effect of biotic and abiotic conditions during the growing period (Cozzolino et al., 2021).

### 3.3 Bioactive properties

Antioxidant properties were evaluated in the hydroethanolic extracts of the studied onion samples based on the TBARS assay (**Table 5**). Our results showed a significant variation, with sample ON2 and ON1 showing the highest and lowest overall activity, while all the samples showed higher activity than Trolox. This finding is in accordance with α-tocopherol content of the studied samples, thus suggesting that this particular compound plays a major role in bioactive properties of the studied onion bulbs. Moreover, Petropoulos et al. (2015) also suggested a varied antioxidant activity among four onion genotypes for the TBARS assay. However, in that study the genotype that recorded the highest antioxidant activity had the lowest α-tocopherol content which suggests that other antioxidant compounds namely polyphenols may also contribute to the overall antioxidant mechanism of plants (Albishi et al., 2013). Moreover, the sample with the highest total phenolic content (ON5) showed lower EC50 values (higher activity) than sample ON3. However, this trend was not consistent among the studied landraces, since sample ON3 recorded higher activity than samples which contained higher amounts of phenols (e.g., On1, ON2 and ON4), which indicates a complex relationship between phenols content and antioxidant activity and the effect that the extraction protocol and the genotype may have in this particular relationship (Gökçe et al., 2010; Nile et al., 2018; Castro-Cegrí et al., 2025). Our findings also suggest that quercetin derivatives were the major polyphenols detected in most of the studied samples (except for ON3) and they could play a key role in the observed antioxidant properties of the studied samples. According to the literature, there is a strong and positive correlation of antioxidant activity with phenolic compounds content and composition in onion bulbs, while quercetin is usually associated with high antioxidant properties due to its capacity to inhibit free radicals and prevent oxidation of biological molecules (Hong et al., 2023; Lee et al., 2025).

**Table 5:** Antioxidant (EC_50_ values, mg/mL) and antibacterial activities (minimal inhibition concentration (MIC) and minimal bactericidal concentration (MBC) expressed in mg/mL) and antifungal activity (MIC and minimal fungicidal concentration (MFC) expressed in mg/mL) of the studied onion hydroethanolic extracts.

	ON1		ON2		ON3		ON4		ON5							
Antioxidant activity											Trolox					
TBARS^*^	1.27 ± 0.06 a		0.28 ± 0.01 b		0.101 ± 0.001 c		0.114 ± 0.002 c		0.092 ± 0.001 c		5.4 ± 0.3					
Antibacterial activity											Streptomicin 1 mg/mL		Methicilin 1 mg/mL		Ampicillin 10 mg/mL	
	MIC	MBC	MIC	MBC	MIC	MBC	MIC	MBC	MIC	MBC	MIC	MBC	MIC	MBC	MIC	MBC
Gram-negative bacteria																
*E. Cloacae*	10	>10	10	>10	10	>10	10	>10	10	>10	0.007	0.007	n.t.	n.t.	0.15	0.15
*E. coli*	5	>10	5	>10	10	>10	2.5	>10	10	>10	0.01	0.01	n.t.	n.t.	0.15	0.15
*P. aeruginosa*	>10	>10	>10	>10	>10	>10	>10	>10	>10	>10	0.06	0.06	n.t.	n.t.	0.63	0.63
*S. enterocolitica*	5	>10	5	>10	10	>10	2.5	>10	10	>10	0.007	0.007	n.t.	n.t.	0.15	0.15
*Y. enterocolitica*	2.5	>10	10	>10	5	>10	10	>10	2.5	>10	0.007	0.007	n.t.	n.t.	0.15	0.15
Gram-positive bacteria																
*B. cereus*	>10	>10	>10	>10	>10	>10	>10	>10	>10	>10	0.007	0.007	n.t.	n.t.	n.t.	n.t.
*L. monocytogenes*	5	>10	10	>10	>10	>10	>10	>10	10	>10	0.007	0.007	n.t.	n.t.	0.15	0.15
*S. aureus*	2.5	>10	5	>10	1.25	>10	2.5	>10	2.5	>10	0.007	0.007	0.007	0.007	0.15	0.15
Antifungal activity	ON1		ON2		ON3		ON4		ON5		Ketoconazole					
	MIC	MFC	MIC	MFC	MIC	MFC	MIC	MFC	MIC	MFC	MIC	MFC				
*A. fumigatus*	10	>10	10	>10	10	>10	10	>10	10	>10	0.5	0.06				
*A.brasiliensis*	10	>10	10	>10	10	>10	10	>10	10	>10	1	0.125				

*Means in the same line followed by different line letters are significantly different according to Duncan’s Multiple Range test (DMRT) at p<0.05. n.t., not tested.

Liguori et al. (2017) also detected significant differences in the antioxidant activity of five white onion landraces determined through the DPPH (2,2-diphenyl-1-picryl-hydrazyl-hydrate) assay, and associated antioxidant activity with phenols content. The study of Metrani et al. (2020) also indicated the importance of the protocol implemented for the assessment of antioxidant activity, since the authors suggested a varied response of the onion extracts to two different assays (e.g. DPPH and ABTS (2,2’-azino-bis(3-ethylbenzothiazoline-6-sulfonic acid)). Similar results have been reported by (Kim et al., 2025) who also recorded different antioxidant capacity of onion skin extracts depending on the extraction protocol. According to Bhandari et al. (2014), the variability of bioactive properties in *Allium* species could be associated with adaptation mechanisms across the cultivation history over the years combined with the artificial selection through vegetative propagation, while Qadir et al. (2017) pointed out the importance of the extraction method and solvent selection for the varied response recorded in antioxidant activity assays.

The antimicrobial properties of the tested onion samples are cited in **Table 5**. The hydroethanolic extracts showed a varied effectiveness against both Gram positive and Gram negative bacteria, while all of them recorded higher MIC and MBC values than the implemented controls. In terms of Gram negative bacteria, ON1 and ON5 sample showed a low inhibitory activity against *Yersinia enterocolitica*, while ON4 was the most effective towards *Escherichia coli* and *Salmonella enterocolitica.* Regarding the Gram-positive bacteria, all the samples were effective towards the Gram positive bacterium *Staphylococcus aureus*, especially ON3 which recorded the lowest overall MIC value (1.25), followed by samples ON1, 4 and 5 with MIC values of 2.5. On the other hand, sample ON1 was the only one that exhibited an inhibitory effect against *Listeria monocytogenes*, whereas the examined hydroethanolic extracts did not demonstrate inhibitory activity against *Bacillus cereus*. Our results did not show significant antifungal activity for any of the studied hydroethanolic extracts towards the tested strains (**Table 5**).

Similarly to our work, Loredana et al. (2019) who tested the antimicrobial properties of two cultivars and two ecotypes of onion cultivated in the Mediterranean basin they suggested a significant inhibitory activity against *S. aureus*, whereas the same authors also reported a high activity towards *B, aureus* and no activity against *E. coli*, a finding that is not aligned with our study. Moreover, Shabir et al. (2022) who performed a literature review regarding the antimicrobial activities of onion peels they also found a varied efficacy against Gram positive and Gram negative for cold water and fresh onion extracts obtained from genotypes with different bulb color. Orǎşan et al. (2017) also reported a varied antimicrobial efficacy of onion formulations depending on the sample preparation (extraction of liquid and lypophilized samples) which affects the content of thiosulfinate compounds in the obtained extracts, while in the study of Anazodo et al. (2024), a significant variation in the antifungal properties of onion extracts was recorded based on the extraction solvent (water, ethanol, methanol and chloroform). Moreover, Kocić-Tanackov et al. (2017) reported a significant funcicidal activity of onion bulbs essential oils against various fungi such as *Aspergillus carbonarius*, *A. wentii*, *A. versicolor*, *Penicillium brevicompactum*, *P. glabrum*, *P. chrysogenum* and *Fusarium* spp., as well as growth inhibition activity *A. niger* and *P. aurantiogriseum*, a finding which further supports the evidence that sulfuric compounds are crucial for bioactive properties of onions. The differences between the literature reports could be associated with differences in genetic material and growing conditions, as well as in the extraction protocol and the solvents used for the extraction, since all these factors may affect the content of phytochemicals such as polyphenols, thiosulfinates and other antioxidants in the obtained extracts and consequently their bioactive properties (Barbu et al., 2023). Therefore, further works are needed to investigate how the extraction protocol, the growing and environmental conditions or the genotype may affect bioactive properties and also associate the phytochemical content of onion bulbs with the observed bioactivities.

Polyphenols such as quercetin derivatives are usually associated with antimicrobial properties in onion extracts (Dhowlaghar et al., 2023; Castro-Cegrí et al., 2025). However, this was not consistent with our results thus suggesting that antioxidant compounds such as polyphenols and α-tocopherol may synergistically contribute to the observed activities. The study of Sim and Nyam (2020) showed that the antimicrobial properties of onion seed oil could be attributed to the synergistic effects of phenolic compounds and tocopherols, although the role of the latter is not well established due to the low content of onion bulbs in such compounds (Petropoulos et al., 2015). Despite that, the variable content of polyphenols and α-tocopherol in our study does not substantiate specific trends in regards to the observed antimicrobial properties. Finally, the lack of antifungal activity observed in our study could be associated with extraction protocol, the genotype and the assay method, since these factors may affect the content of antifungal compounds and the susceptibility of fungal strains to onion extracts (Vazquez-Armenta et al., 2015; Mardani et al., 2023).

## 4 Conclusions

The present work reports for the first time the chemical composition of four Greek onion landraces collected from two distinct localities in comparison with a commercially available cultivar. Our results indicate a great variability in nutritional value and chemical profile of the studied genotypes, while varied bioactivities were also recorded. Interestingly, the local landrace with elliptical bulbs from Thiva region (sample ON5) showed the largest content of polyphenols and quercetin in particular, which was accompanied by higher antioxidant activity. On the other hand, the commercial cultivar was richer in total and individual free sugars, as well as in total dietary fibers and total organic acids which indicate a sweeter taste and higher pungency. In conclusion, the great variability in the chemical composition and bioactivities among the studied genotypes shows the high potential of the underexplored genetic material which so far is preserved at a local level and its further valorizing via breeding programs for the selection of improved genotypes with enhanced quality and functional features. However, further studies under specified agronomic practices (e.g., fertilization and irrigation regime) and soil conditions are needed to further investigate the impact of pre-harvest factors on the quality of bulbs and how the specific conditions of each region contribute to the distinctive traits of each landrace.

## Data Availability

The raw data supporting the conclusions of this article will be made available by the authors, without undue reservation.
